# Characterization of multidrug-resistant *Acinetobacter baumannii* strain ATCC BAA1605 using whole-genome sequencing

**DOI:** 10.1186/s13104-021-05493-z

**Published:** 2021-03-04

**Authors:** Kah Ern Ten, Muhammad Zarul Hanifah Md Zoqratt, Qasim Ayub, Hock Siew Tan

**Affiliations:** 1 School of Science, Monash University Malaysia, 47500 Bandar Sunway, Selangor Darul Ehsan Malaysia; 2 Monash University Malaysia Genomics Facility, 47500 Bandar Sunway, Selangor Darul Ehsan Malaysia; 3 Tropical Medicine and Biology Multidisciplinary Platform, 47500 Bandar Sunway, Selangor Darul Ehsan Malaysia

**Keywords:** *Acinetobacter baumannii*, Antimicrobial resistance, Crispr/cas system, Hybrid genome assembly, Nanopore MinION, Illumina miSeq

## Abstract

**Objective:**

The nosocomial pathogen, *Acinetobacter baumannii,* has acquired clinical significance due to its ability to persist in hospital settings and survive antibiotic treatment, which eventually resulted in the rapid spread of this bacterium with antimicrobial resistance (AMR) phenotypes. This study used a multidrug-resistant *A. baumannii* (strain ATCC BAA1605) as a model to study the genomic features of this pathogen.

**Results:**

One circular chromosome and one circular plasmid were discovered in the complete genome of *A. baumannii* ATCC BAA1605 using whole-genome sequencing. The chromosome is 4,039,171 bp long with a GC content of 39.24%. Many AMR genes, which confer resistance to major classes of antibiotics (beta-lactams, aminoglycosides, tetracycline, sulphonamides), were found on the chromosome. Two genomic islands were predicted on the chromosome, one of which (Genomic Island 1) contains a cluster of AMR genes and mobile elements, suggesting the possibility of horizontal gene transfer. A subtype I-F CRISPR-Cas system was also identified on the chromosome of *A. baumannii* ATCC BAA1605.

This study provides valuable genome data that can be used as a reference for future studies on *A. baumannii.* The genome of *A. baumannii* ATCC BAA1605 has been deposited at GenBank under accession no. CP058625 and CP058626.

## Introduction

*Acinetobacter baumannii*, a gram-negative opportunistic human pathogen, has currently been recognized as one of the most challenging nosocomial pathogens. Ventilator-associated-pneumonia (VAP) is the major outcome of *A. baumannii* infections, with an overall in-hospital mortality rate of 63.3% [1]. Other presentations may include meningitis, urinary tract infection, bacteremia and skin or wound infections [2]. Multidrug-resistant (MDR) *A. baumannii* was thought to have first emerged in the United States of America in military treatment facilities during the 2003–2004 outbreak MDR *A. baumannii* which caused serious infections to the injured military personnel returned from the war. This subsequently resulted in the spread of MDR *A. baumannii* to other areas and increased the rate of distribution of resistance genes [3]. Carbapenem-resistant *A. baumannii* has recently been categorized by the World Health Organization (WHO) as a top priority pathogen requiring urgent development of novel antibiotics [4]. Several studies have reported a higher than the national average (50–60%) carbapenem resistance rates of *A. baumannii* isolates from individual hospitals in Malaysia. For example, carbapenem resistance rate of > 70% was reported for the clinical isolates collected from Universiti Kebangsaan Malaysia Medical Centre (UKMCC Kuala Lumpur, Malaysia) between 2010 and 2011 [5]. Resistance to other major classes of antibiotics (such as cephalosporins, aminoglycosides, fluoroquinolones, etc.) was also reported in Malaysia [6].

*A. baumannii* tolerates unfavourable environmental conditions, such as nutrient limitation and desiccation. It colonizes almost any surfaces including medical equipment. The ability of *A. baumannii* to form biofilm enhances its survival under stress conditions [7]. This could increase the chances of transmission and MDR development attained by either mutations or genetic elements. Clustered regularly interspaced short palindromic repeats and their associated Cas proteins (CRISPR-Cas) is a responsive immune system that could play a role in the exchange of bacterial genetic information, colonization and biofilm production [8]. CRISPR-Cas system can be classified into two main classes, which includes 6 major types and 33 different subtypes [9]. Subtype I-Fb is reported as the most common CRISPR-Cas in *A. baumannii* [10].

Understanding the genome characteristics of *A. baumannii*, especially the MDR strains, could provide useful information in dealing with this pathogen such as drug development. In this study, we aimed to sequence the MDR *A. baumannii* strain ATCC BAA1605 which was originally isolated from a sputum sample of military personnel returning from Afghanistan and admitted to a Canadian hospital in 2006. This strain has been known for its MDR phenotype; however, its genomic features remain poorly studied. To our knowledge, this is the first reported complete genome of *A. baumannii* strain ATCC BAA1605.

## Materials and methods

### Sample preparation

*A. baumannii* ATCC BAA1605 (ATCC® BAA-1605™) was purchased from American Type Culture Collection (ATCC, USA). This strain was originally isolated from a sputum sample of a hospitalized patient according to ATCC (USA). *A. baumannii* was cultured on Mueller–Hinton broth (Oxoid, UK) and incubated overnight at 37℃ with continuous shaking at 200 rpm. DNA was isolated using phenol–chloroform phase-separation method according to Sambrook and Russell [11]. Leeds *Acinetobacter* medium (LAM) supplemented with antibiotics (HiMedia, India) was used to selectively isolate MDR *Acinetobacter*. Selective supplement composed of 3 antibiotics: vancomycin, cefsulodin and cefradine. Antibiotic-sensitive *A. baumannii* strain 65 (isolated from Segamat, Malaysia, unpublished data) was used as a control. Antibiotic susceptibility test (AST) was performed using disk diffusion method according to Clinical and Laboratory Standard Institute (CLSI) [12]. Classes of antibiotics tested were listed in Additional file 1: Figure S2C.

### De novo whole-genome sequencing and assembly

A hybrid short- and long-read based-WGS was performed to construct the complete genome of *A. baumannii* ATCC BAA1605. Briefly, short-read sequencing data was generated by Nextera XT library preparation kit and sequenced on the Illumina Miseq using a 2 × 250 bp paired-end configuration. DNA libraries for long-reads sequencing were prepared using the Ligation Sequencing Kit protocol (SQK-LSK109) and long-reads sequencing data was generated on a MinION FLO-MIN106 flow cell and MinION MK1B sequencing device (Oxford Nanopore Technologies). Base-calling was conducted using Guppy v3.2.10 through MinKnow v3.6.17, using fast base calling configuration. Quality of short Illumina reads was assessed using FastQC v0.11.5 (https://github.com/s-andrews/FastQC), followed by adapter trimming using Trimmomatic v0.36 [13]. Chromosomal assembly was performed using Flye v2.7 [14]. Plasmid recovery was done by checking short-reads sequencing data assembled de novo using SPAdes v3.13.0 [15]. The whole-genome sequence was later corrected and polished using Pilon v1.23 [16]. Quality of the corrected assembly was evaluated using BUSCO v4.0.6 [17], using pseudomonales_odb10 as database.

### Genome annotation, genome map and plasmid identification

The whole genome was annotated using Prokka v1.13 [18]. Genome map was plotted using BLAST Ring Image Generator (BRIG) v0.95 [19]. Plasmid identification was determined using BLASTn [20] against NCBI database, and sourmash v3.3.0 search-containment method [21] against PLSDB database [22]. Top 10 plasmids from sourmash results were aligned with *A. baumannii* ATCC BAA1605 plasmid (CP058626) using Mauve v2.4.0 progressive alignment [23].

### Genome analysis

Whole genome of *A. baumannii* ATCC BAA1605 was compared with two reference strains: *A. baumannii* ATCC BAA-1790 (the only *A. baumannii* ATCC BAA strain with complete genome available in NCBI database) and *A. baumannii* ASM211692v1 (the representative strain of *A. baumannii* in NCBI). Whole genome alignment of the 3 strains was constructed using Mauve v2.4.0 progressive alignment [23]. AMR genes were identified by Abricate v1.0.1 (https://github.com/tseemann/abricate), using Comprehensive Antibiotic Resistance Database (CARD) [24]. Prophage regions and CRISPR-Cas proteins were detected using the web-tools, PHASTER [25] and CRISPRCasFinder [26], respectively. CRISPR-Cas with evidence levels of 3 and 4 represented highly likely candidates according to Couvin, Bernheim [26], and was selected for further analysis. Core genomes of different strains of *A. baumannii* that carry CRISPR-Cas (downloaded from the NCBI database) were extracted using Roary v3.13.0 [27]. The phylogenetic tree of CRISPR-Cas of *A. baumannii* was constructed with the concatenated core genome sequences using FastTree [28] and visualized using FigTree v1.4.3 (http://tree.bio.ed.ac.uk/software/figtree/). Genomic islands were predicted using IslandViewer 4 [29] and schematic representation of the genes was constructed using DNA Features Viewer v3.0.1 [30].

### Genome accession numbers

The SRA accession numbers of long- and short-reads sequence data are SRX8666155 and SRX8666156, respectively. The sequences of the complete annotated genome of *A. baumannii* ATCC BAA1605 has been deposited at GenBank with accession CP058625 (https://www.ncbi.nlm.nih.gov/nuccore/CP058625.1) and CP058626 (https://www.ncbi.nlm.nih.gov/nuccore/CP058626.1).

## Results

### Genome properties

**Fig. 1:**
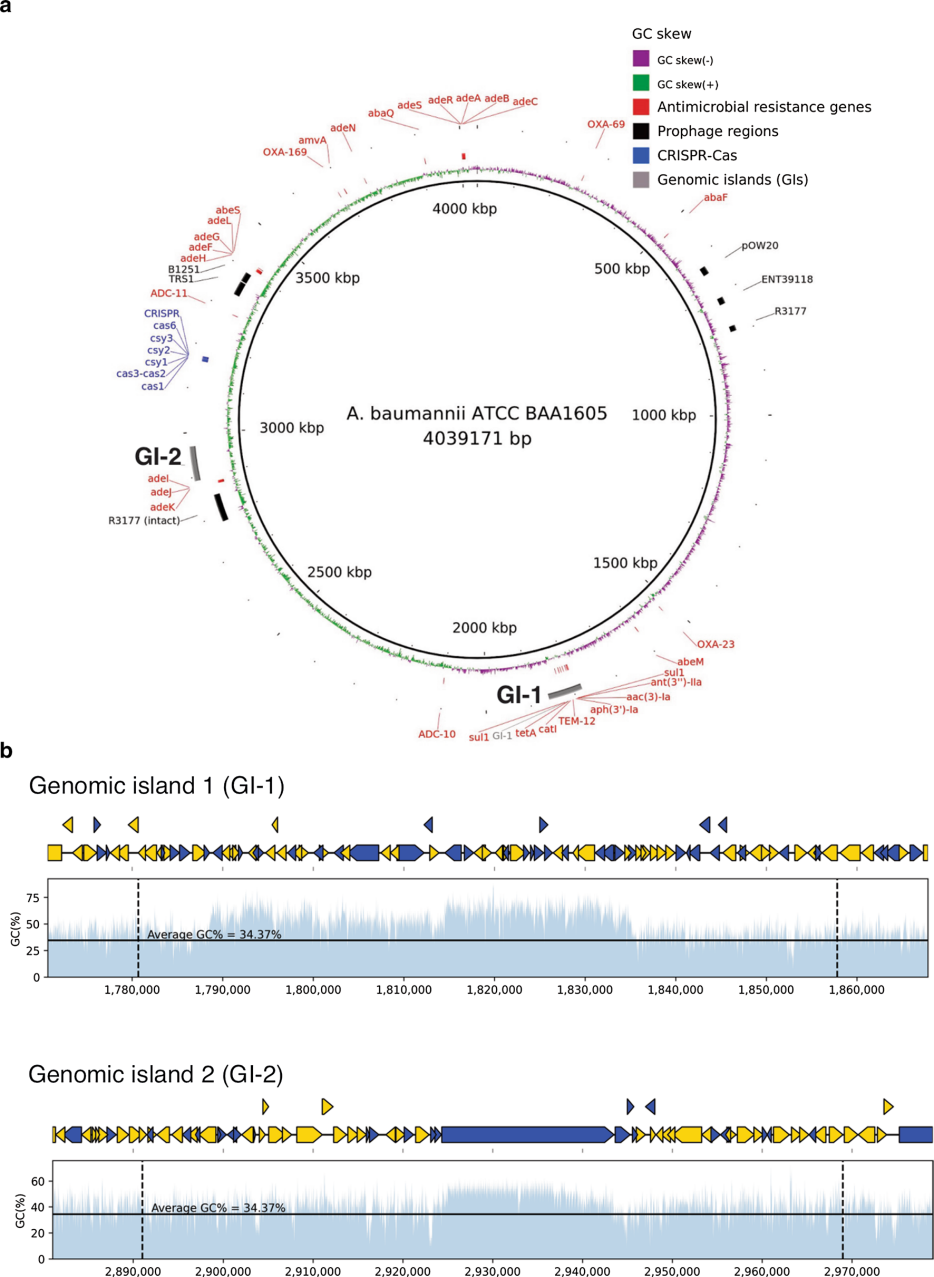
Genomic features of *A. baumannii* ATCC BAA1605 chromosome (CP058625). **a** Genome map. The innermost colored circle denotes the GC skew of genomic sequences (purple: negative; green: positive), followed by distributions of antimicrobial resistance genes (red), prophage regions (black), CRISPR-Cas system (blue) and genomic islands (GIs) predicted by IslandViewer 4 (grey). **b** Schematic representation of the genes present in the predicted GIs from IslandViewer 4 and their GC contents. The upper panel depicts the genes and their GC contents found in the GI-1 (approximately 1.78 Mb to 1.86 Mb), while lower panel depicts the genes and their GC contents found in the GI-2 (approximately 2.89 Mb to 2.97 Mb). Yellow arrows: Annotated CDS; Blue arrows: Unannotated/hypothetical proteins

*A. baumannii* ATCC BAA1605 contains one circular chromosome (CP058625) and one circular plasmid (CP058626) with the sizes of 4,039,171 bp (GC content = 39.24%) and 8,731 bp (GC content = 34.37%), respectively (Fig. 1a, Additional file 1: Table S1). A high degree of completeness was obtained for this genome with a BUSCO score of 98.9%, of which 774 genes were complete, 6 were fragmented, and 2 were missing BUSCO orthologs out of the 782 BUSCO groups searched. Genome annotation is summarized in Table S2 and Table S3. Comparing to the two reference strains (*A. baumannii* ATCC BAA-1790 and *A. baumannii* ASM211692v1), *A. baumannii* ATCC BAA1605 has a slightly larger number of genes and chromosome size but carry a smaller plasmid (Additional file 1: Table S4). The chromosomes and plasmids for the three strains share similar GC content. Whole genome alignments of the 3 strains showed some genome rearrangement (Additional file 1: Figure S1).

### Antimicrobial resistance

Thirty AMR genes were detected on the chromosome of *A. baumannii* ATCC BAA1605 and their distribution is shown in Fig. 1a. No AMR genes were detected on the plasmid. Identification of these AMR genes suggests the resistance of this pathogen to few major classes of antibiotics, including beta-lactams, fluoroquinolone, tetracycline, aminoglycoside, and sulphonamide (Additional file 1: Table S5). *A. baumannii* ATCC BAA1605 grew on LAM supplemented with antibiotics that select for MDR *Acinetobacter* (Additional file 1: Figure S2A and S2B). This finding is further supported by the results of AST (Additional file 1: Figure S2C), where *A. baumannii* ATCC BAA1605 showed resistance to all classes of antibiotics tested.

### Prophage regions and genomic islands

One intact prophage region was identified on the *A. baumannii* ATCC BAA1605 chromosome with a length of 65.5 Kb (Fig. 1a, Additional file 1: Table S6). There are 79 open reading frames (ORFs) present in this intact prophage region. The best hit for this region corresponds to the *Acinetobacter* phage YMC11/11/R3177. There are five additional incomplete prophage regions scattered throughout the genome (Fig. 1a, Additional file 1: Table S6).

Two clear genomic islands (GI-1 and GI-2) were identified on the chromosome of this bacterium (Fig. 1a). Genes found within these GIs are listed in Additional file 1: Table S7 and Table S8. A cluster of AMR genes, such as *bla* and *tet*, and DNA recombination enzymes including resolvase (*tnpR*) and DNA-invertase (*hin)* were found in GI-1. The GC content in these GIs is generally higher than the average GC content of the genome (34.37%) (Fig. 1b).

### Plasmid identification and verification

**Fig. 2:**
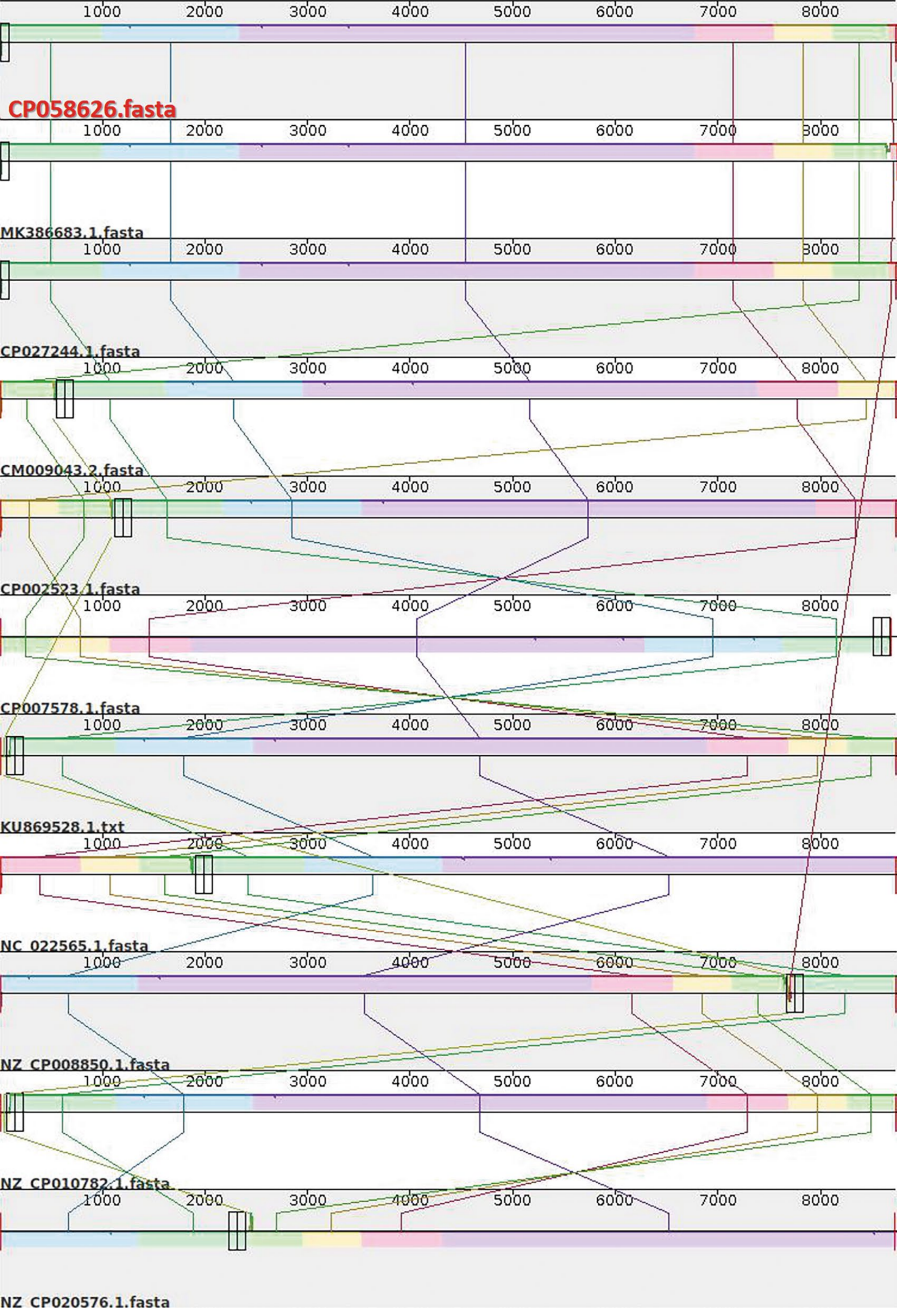
Plasmid sequence alignments of *A. baumannii* ATCC BAA1605 plasmid (CP058626) with top 10 plasmids from sourmash results using Mauve. The plasmid of *A. baumannii* ATCC BAA1605 strain used in this study is shown on top and highlighted in red. Each coloured blocks depicts the homologous sites of sequence that aligned to part of another genome

Plasmid sequence of *A. baumannii* ATCC BAA1605 was aligned with the top 10 plasmids obtained from sourmash results (Additional file 1: Table S9) and shown in Fig. 2. BLASTn results revealed 100% identity and coverage of *A. baumannii* ATCC BAA1605 plasmid with the plasmids of other *A. baumannii* strains in the NCBI database. Comparison against PLSDB database using sourmash showed 100% similarity with *A. baumannii* strain ABAY14012 plasmid pABAY14012_4D (MK386683.1), followed by *A. baumannii* strain WCHAB005078 plasmid p2_005078 (CP027244.1) with a similarity of 99.8% (Fig. 2, Additional file 1: Table S9).

### CRISPR-Cas system

**Fig. 3:**
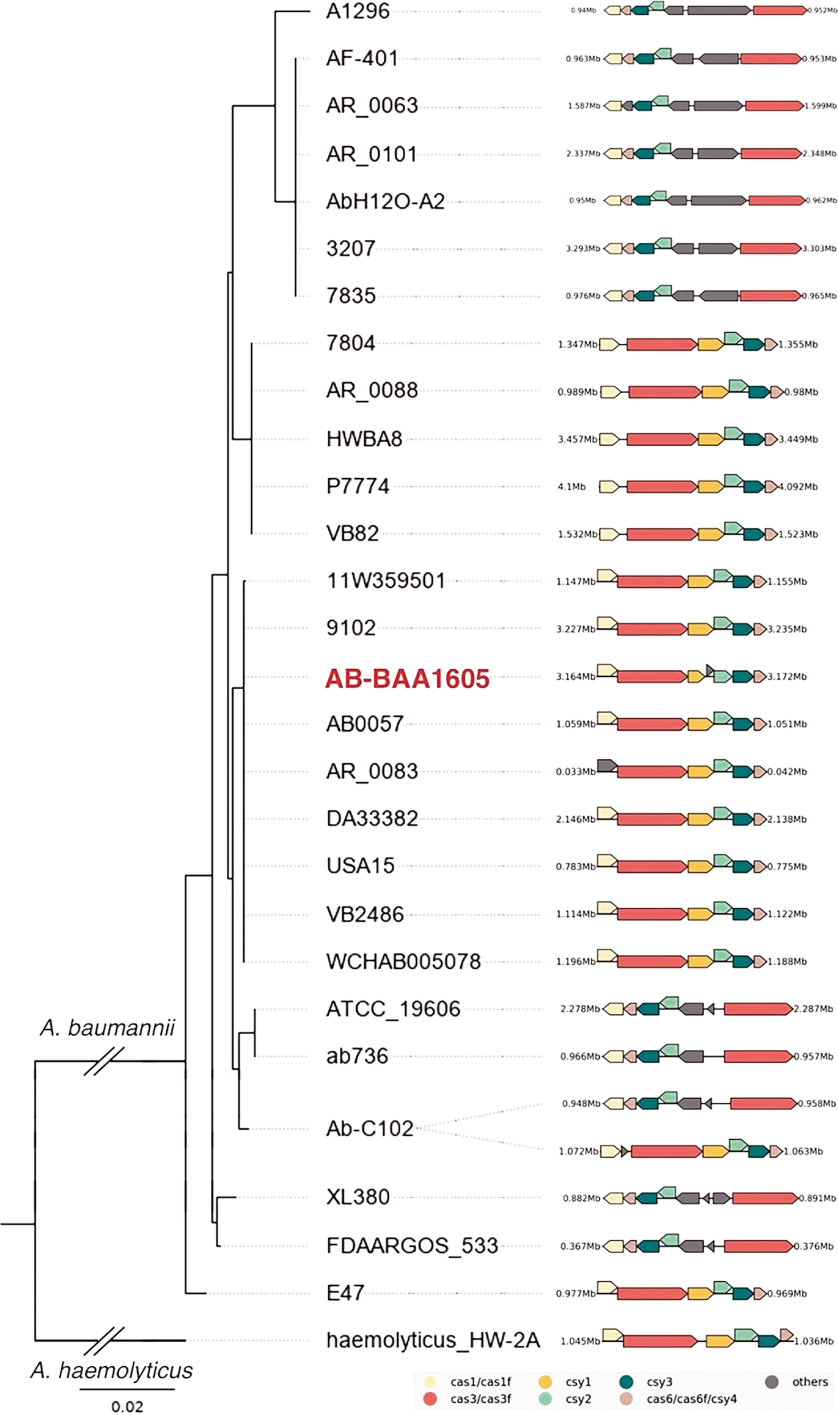
Phylogenetic tree of CRISPR-Cas system of different strains of *A. baumannii*. Organization of the CRISPR-Cas systems in the genomes are shown on the right. Yellow, *cas1/cas1f*; Red, *cas3/cas3f*; Orange, *csy1*; Light green, *csy2*; Dark green, *csy3*; Brown, *cas6/cas6f/cas4*; Gray, CDS not related to CRISPR. The *A. baumannii* ATCC BAA1605 strain used in this study is highlighted in red. *A. haemolyticus* strain HW-2A with CRISPR-Cas system was used as an outgroup

A subtype I-F CRISPR-Cas was identified on the chromosome of *A. baumannii* ATCC BAA1605, with a 3150 bp CRISPR array containing 52 spacers (Fig. 3, Additional file 1: Table S10). The spacers are flanked by 28 bp CRISPR repeats. The Cas proteins are listed in Additional file 1: Table S11. Phylogenetic tree of *A. baumannii* carrying CRISPR-Cas was shown in Fig. 3. It is observed that strains with close phylogenetic relationships have similar genes and share gene synteny on their CRISPR-Cas system.

## Discussion

WGS reveals one circular chromosome and one circular plasmid in this MDR *A. baumannii* ATCC BAA1605. High degree of completeness from BUSCO score indicates successful genome assembly and accurate analysis. There are many AMR genes identified on the chromosome of this pathogen, indicating its resistance to a few major classes of antibiotics. This has been further evidenced by culturing *A. baumannii* ATCC BAA1605 on LAM added with selective supplement that contained antibiotics selectively for MDR *Acinetobacter*, and AST results. For example, multiple *bla* genes that encode carbapenem-hydrolyzing class D β-lactamases, including the highly prevalent *bla*_*OXA-23*_ gene [31], were detected on the chromosome of this pathogen, suggested its resistance to carbapenems which then confirmed by AST. One intact, complete prophage region was detected on the chromosome with high similarity to *Acinetobacter* phage YMC11/11/R3177. Genes, such as prophage integrase *intA* and *intS*, and virulence regulon transcriptional activator *virF*, were found within the intact prophage region, suggesting their roles in regulating many phage-encoded virulence factors.

GI-1 that was detected on the chromosome contains a cluster of AMR genes and mobile genetic elements. This could suggest the possibility of acquired AMR via horizontal gene transfer since no AMR genes were found on the plasmid. It is reported that the genomic islands of pathogenic *A. baumannii* generally possess genes such as heavy metal resistance genes, AMR genes and competence proteins, that facilitate their survival under unfavourable conditions [32]. The findings of this study are consistent with this notion.

A subtype I-F CRISPR-Cas system was identified on the chromosome of *A. baumannii* ATCC BAA1605. Since the spacers in the CRISPR array do not change over time, the high number of spacers identified in the CRISPR loci of this pathogen suggests that it might have encountered a high number of phages attacks. The presence of CRISPR-Cas system could explain the low number of plasmid found within this genome.

## Conclusion

Multidrug-resistant *A. baumannii* has become an emerging threat to public health, especially to immunocompromised patients. Yet, there are many drug-resistant *A. baumannii* that have not been fully explored in terms of their genomes. This is the first reported complete genome of a MDR *A. baumannii* (strain ATCC BAA1605). This study provides data that can be used as a reference for future studies on MDR *A. baumannii* and improves our understanding of the genomic features of this reference strain.

### Limitations

The virulent determinants identified in the genome of *A. baumannii* ATCC BAA1605 could serve as preliminary data; however, future experiments, such as PCR, can be conducted to validate the presence of these virulent genes.

## Supplementary Information

**Additional file 1: Table S1.** Summary of the genome of *A. baumannii* ATCC BAA1605: one chromosome and one plasmid. **Table S2.** Summary of annotation of *A. baumannii* ATCC BAA1605 chromosome using Prokka. **Table S3.** Summary of annotation of *A. baumannii* ATCC BAA1605 plasmid using Prokka. **Table S4.** Comparisons of the chromosome and plasmid of *A. baumannii* strain ATCC BAA1605 with *A. baumannii* strain ATCC BAA-1790 and *A. baumannii* ASM211692v1. **Table S5.** Antibiotic resistance profiles of *A. baumannii* ATCC BAA1605 identified by CARD. **Table S6.** Predicted prophage regions in *A. baumannii* ATCC BAA1605 using PHASTER. **Table S7.** Genes found in Genomic Island 1 (1.78 Mb to 1.86 Mb) predicted by IslandViewer 4 in *A. baumannii* ATCC BAA1605 and their coordinates. **Table S8**. Genes found in Genomic Island 2 (2.89 Mb to 2.97 Mb) predicted by IslandViewer 4 in *A. baumannii* ATCC BAA1605 and their coordinates. **Table S9.** Top 10 plasmids and their similarity with *A. baumannii* ATCC BAA1605 plasmid using sourmash search-containment method against PLSDB database. **Table S10.** Summary of CRISPR array identified in chromosome of *A. baumannii* ATCC BAA1605. **Table S11.** Subtype I-F *cas* genes identified in the chromosome of *A. baumannii* ATCC BAA1605 and their coordinates. **Figure S1.** Whole genome alignments of *A. baumannii* ATCC BAA1605 (top), *A. baumannii* ATCC BAA-1790 (middle) and *A. baumannii* ASM211692v1 (bottom) using Mauve. Each coloured blocks depicts the homologous sites of sequence that aligned to part of another genomes. **Figure S2.** Identification and antibiotic resistance profiling of *A. baumannii* ATCC BAA1605. MDR *A. baumannii* ATCC BAA1605 and antibiotic-sensitive *A. baumannii* strain 65 (control) cultured on Leeds *Acinetobacter* medium either without selective supplement (A) or with antibiotics supplementation (B). (C) Antibiotic susceptibility profiling of *A. baumannii* ATCC BAA1605 using disk diffusion method.

## Data Availability

The complete sequences of the genome of *A. baumannii* ATCC BAA1605 are available in GenBank with the accession numbers: CP058625 and CP058626 (Bioproject ID: PRJNA643902, https://www.ncbi.nlm.nih.gov/bioproject/PRJNA643902/).

## Abbreviations

AMR: Antimicrobial resistance
Cas: CRISPR-associated protein
CDS: Coding sequence
CRISPR: Clustered regularly interspaced short palindromic repeats
GI: Genomic Island
MDR: Multidrug-resistant
ORF: Open reading frame
WGS: Whole-genome sequencing
PCR: Polymerase chain reaction
LAM: Leeds *Acinetobacter* medium

## References

[1] Čiginskienė A, Dambrauskienė A, Rello J, Adukauskienė D (2019). Ventilator-associated pneumonia due to drug-resistant *Acinetobacter baumannii*: risk factors and mortality relation with resistance profiles, and independent predictors of in-hospital mortality. Medicina.

[2] Bergogne-Berezin E, Towner K (1996). Acinetobacter spp. as nosocomial pathogens: microbiological, clinical, and epidemiological features. Clin Microbiol Rev..

[3] Scott P, Deye G, Srinivasan A, Murray C, Moran K, Hulten E (2007). An outbreak of multidrug-resistant *Acinetobacter baumannii-calcoaceticus* complex infection in the US military health care system associated with military operations in Iraq. Clin Infect Dis.

[4] Tacconelli E, Carrara E, Savoldi A, Harbarth S, Mendelson M, Monnet DL (2018). Discovery, research, and development of new antibiotics: the WHO priority list of antibiotic-resistant bacteria and tuberculosis. Lancet Infect Dis.

[5] Biglari S, Alfizah H, Ramliza R, Rahman MM (2015). Molecular characterization of carbapenemase and cephalosporinase genes among clinical isolates of *Acinetobacter baumannii* in a tertiary medical centre in Malaysia. J Med Microbiol.

[6] Mohd Rani F, Rahman NIA, Ismail S, Alattraqchi AG, Cleary DW, Clarke SC (2017). Acinetobacter spp. infections in Malaysia: a review of antimicrobial resistance trends, mechanisms and epidemiology. Front Microbiol..

[7] Gaddy JA, Actis LA (2009). Regulation of *Acinetobacter baumannii* biofilm formation. Future Microbiol.

[8] Shah SA, Garrett RA (2011). CRISPR/Cas and Cmr modules, mobility and evolution of adaptive immune systems. Res Microbiol.

[9] Koonin EV, Makarova KS, Zhang F (2017). Diversity, classification and evolution of CRISPR-Cas systems. Curr Opin Microbiol.

[10] Karah N, Samuelsen O, Zarrilli R, Sahl J, Wai S, Uhlin B (2015). CRISPR-Cas subtype I-Fb in *Acinetobacter baumannii*: evolution and utilization for strain subtyping. PLoS ONE.

[11] Sambrook J, Russell DW (2006). Purification of nucleic acids by extraction with phenol:chloroform. CSH Protoc..

[12] CLSI. Performance standards for antimicrobial susceptibility testing. 28th ed. CLSI supplement M100. Wayne, PA: Clinical and Laboratory Standards Institute; 2018.

[13] Bolger AM, Lohse M, Usadel B (2014). Trimmomatic: a flexible trimmer for Illumina sequence data. Bioinformatics (Oxford, England).

[14] Kolmogorov M, Yuan J, Lin Y, Pevzner PA (2019). Assembly of long, error-prone reads using repeat graphs. Nat Biotechnol.

[15] Bankevich A, Nurk S, Antipov D, Gurevich AA, Dvorkin M, Kulikov AS (2012). SPAdes: a new genome assembly algorithm and its applications to single-cell sequencing. J Comput Biol.

[16] Walker BJ, Abeel T, Shea T, Priest M, Abouelliel A, Sakthikumar S (2014). Pilon: an integrated tool for comprehensive microbial variant detection and genome assembly improvement. PLoS ONE.

[17] Simão FA, Waterhouse RM, Ioannidis P, Kriventseva EV, Zdobnov EM (2015). BUSCO: assessing genome assembly and annotation completeness with single-copy orthologs. Bioinformatics.

[18] Seemann T (2014). Prokka: rapid prokaryotic genome annotation. Bioinformatics.

[19] Alikhan N-F, Petty NK, Ben Zakour NL, Beatson SA (2011). BLAST Ring Image Generator (BRIG): simple prokaryote genome comparisons. BMC Genomics.

[20] Altschul SF, Gish W, Miller W, Myers EW, Lipman DJ (1990). Basic local alignment search tool. J Mol Biol.

[21] Pierce NT, Irber L, Reiter T, Brooks P, Brown CT (2019). Large-scale sequence comparisons with sourmash. F1000Res.

[22] Galata V, Fehlmann T, Backes C, Keller A (2019). PLSDB: a resource of complete bacterial plasmids. Nucleic Acids Res.

[23] Darling ACE, Mau B, Blattner FR, Perna NT (2004). Mauve: multiple alignment of conserved genomic sequence with rearrangements. Genome Res.

[24] Jia B, Raphenya AR, Alcock B, Waglechner N, Guo P, Tsang KK (2017). CARD 2017: expansion and model-centric curation of the comprehensive antibiotic resistance database. Nucleic Acids Res.

[25] Arndt D, Grant JR, Marcu A, Sajed T, Pon A, Liang Y (2016). PHASTER: a better, faster version of the PHAST phage search tool. Nucleic Acids Res.

[26] Couvin D, Bernheim A, Toffano-Nioche C, Touchon M, Michalik J, Néron B (2018). CRISPRCasFinder, an update of CRISRFinder, includes a portable version, enhanced performance and integrates search for Cas proteins. Nucleic Acids Res.

[27] Page AJ, Cummins CA, Hunt M, Wong VK, Reuter S, Holden MTG (2015). Roary: rapid large-scale prokaryote pan genome analysis. Bioinformatics.

[28] Price MN, Dehal PS, Arkin AP (2009). FastTree: computing large minimum evolution trees with profiles instead of a distance matrix. Mol Biol Evol.

[29] Bertelli C, Laird MR, Williams KP, Lau BY, Hoad G, Winsor GL (2017). IslandViewer 4: expanded prediction of genomic islands for larger-scale datasets. Nucleic Acids Res.

[30] Zulkower V, Rosser S (2020). DNA Features Viewer: a sequence annotation formatting and plotting library for Python. Bioinformatics.

[31] Mugnier PD, Poirel L, Naas T, Nordmann P (2010). Worldwide dissemination of the *blaOXA-23* carbapenemase gene of *Acinetobacter baumannii*. Emerg Infect Dis.

[32] Yakkala H, Samantarrai D, Gribskov M, Siddavattam D (2019). Comparative genome analysis reveals niche-specific genome expansion in Acinetobacter baumannii strains (Research Article) (Report). PLoS ONE.

