# Supplementary material for: Characterization of multidrug-resistant Acinetobacter baumannii strain ATCC BAA1605 using whole-genome sequencing
Source: BMC Res Notes. 2021 Mar 4;14:83. doi: 10.1186/s13104-021-05493-z (PMC7934414; doi:10.1186/s13104-021-05493-z)
Supplement: Supplementary file 1 — Additional file 1: Table S1. Summary of the genome of A. baumannii ATCC BAA1605: one chromosome and one plasmid. Table S2. Summary of annotation of A. baumannii ATCC BAA1605 chromosome using Prokka. Table S3. Summary of annotation of A. baumannii ATCC BAA1605 plasmid using Prokka. Table S4. Comparisons of the chromosome and plasmid of A. baumannii strain ATCC BAA1605 with A. baumannii strain ATCC BAA-1790 and A. baumannii ASM211692v1. Table S5. Antibiotic resistance profiles of A. baumannii ATCC BAA1605 identified by CARD. Table S6. Predicted prophage regions in A. baumannii ATCC BAA1605 using PHASTER. Table S7. Genes found in Genomic Island 1 (1.78 Mb to 1.86 Mb) predicted by IslandViewer 4 in A. baumannii ATCC BAA1605 and their coordinates. Table S8. Genes found in Genomic Island 2 (2.89 Mb to 2.97 Mb) predicted by IslandViewer 4 in A. baumannii ATCC BAA1605 and their coordinates. Table S9. Top 10 plasmids and their similarity with A. baumannii ATCC BAA1605 plasmid using sourmash search-containment method against PLSDB database. Table S10. Summary of CRISPR array identified in chromosome of A. baumannii ATCC BAA1605. Table S11. Subtype I-F cas genes identified in the chromosome of A. baumannii ATCC BAA1605 and their coordinates. Figure S1. Whole genome alignments of A. baumannii ATCC BAA1605 (top), A. baumannii ATCC BAA-1790 (middle) and A. baumannii ASM211692v1 (bottom) using Mauve. Each coloured blocks depicts the homologous sites of sequence that aligned to part of another genomes. Figure S2. Identification and antibiotic resistance profiling of A. baumannii ATCC BAA1605. MDR A. baumannii ATCC BAA1605 and antibiotic-sensitive A. baumannii strain 65 (control) cultured on Leeds Acinetobacter medium either without selective supplement (A) or with antibiotics supplementation (B). (C) Antibiotic susceptibility profiling of A. baumannii ATCC BAA1605 using disk diffusion method. [file 13104_2021_5493_MOESM1_ESM.docx]

# Supplementary Materials

**Characterization of Multidrug-Resistant *Acinetobacter baumannii* strain ATCC BAA1605 using Whole-Genome Sequencing**

Kah Ern Ten^1^, Muhammad Zarul Hanifah Md Zoqratt^1, 2^, Qasim Ayub^1, 2^, Hock Siew Tan^1, 3*^

^1^ School of Science, Monash University Malaysia, 47500 Bandar Sunway, Selangor Darul Ehsan, Malaysia

^2^ Monash University Malaysia Genomics Facility, 47500 Bandar Sunway, Selangor Darul Ehsan, Malaysia

^3^ Tropical Medicine and Biology Multidisciplinary Platform, 47500 Bandar Sunway, Selangor Darul Ehsan, Malaysia

*Corresponding author: Tan Hock Siew, tan.hocksiew@monash.edu

| **Label** | **Size (bp)** | **Topology** | **GC content (%)** | **Accession** |
| --- | --- | --- | --- | --- |
| Chromosome 1 | 4039171 | Circular | 39.24 | CP058625 |
| Plasmid 1 | 8731 | Circular | 34.37 | CP058626 |

**Table S1**. Summary of the genome of *A. baumannii* ATCC BAA1605: one chromosome and one plasmid.

| Number of contigs | 1 |
| --- | --- |
| Bases | 4039171 |
| Coding sequence | 3898 |
| Gene | 4030 |
| Transfer-messenger RNA | 1 |
| Ribosomal RNA | 18 |
| Transfer RNA | 75 |
| Miscellaneous RNA | 38 |

**Table S2**. Summary of annotation of *A. baumannii* ATCC BAA1605 chromosome using Prokka.

| Number of contigs | 1 |
| --- | --- |
| Bases (bp) | 8731 |
| Coding Sequence | 12 |
| Gene | 12 |

**Table S3**. Summary of annotation of *A. baumannii* ATCC BAA1605 plasmid using Prokka.

| **Strains** | **Chromosome** | | | **Plasmid** | | |
| --- | --- | --- | --- | --- | --- | --- |
|  | **Size (bp)** | **GC (%)** | **Number of Genes** | **Size (bp)** | **GC (%)** | **Number of Genes** |
| ATCC BAA1605 | 4039171 | 39.2 | 4030 | 8731 | 34.4 | 12 |
| ATCC BAA-1790 | 3963235 | 39.2 | 3799 | 67023 | 33.4 | 86 |
| ASM211692v1 | 3980886 | 39.2 | 3798 | 9539 | 34.7 | 12 |

**Table S4.** Comparisons of the chromosome and plasmid of *A. baumannii* strain ATCC BAA1605 with *A. baumannii* strain ATCC BAA-1790 and *A. baumannii* ASM211692v1.

| **Antibiotic class** | **Resistance gene** | **Predicted resistance** |
| --- | --- | --- |
| Aminoglycoside | *ANT(3'')-IIa* | Aminoglycoside |
|  | *AAC(3)-Ia* | Aminoglycoside |
|  | *APH(3')-Ia* | Aminoglycoside |
| Beta-lactam | *blaOXA-69* | Cephalosporin, penam |
|  | *blaOXA-23* | Cephalosporin, penam |
|  | *blaTEM-12* | Cephalosporin, monobactam, penam, penem |
|  | *blaADC-10* | Cephalosporin |
|  | *blaADC-11* | Cephalosporin |
|  | *blaOXA-169* | Cephalosporin, penam |
|  | *adeN* | Carbapenem, cepholosporin, diaminopyrimidine, fluoroquinolone, lincosamide, macrolide, penem, phenicol, rifamycin, tetracycline |
|  | *adeK* | Carbapenem, cephalosporin, diaminopyrimidine, fluoroquinolone, lincosamide, macrolide, penem, phenicol, rifamycin, tetracycline |
|  | *adeJ* | Carbapenem, cephalosporin, diaminopyrimidine, fluoroquinolone, lincosamide, macrolide, penem, phenicol, rifamycin, tetracycline |
|  | *adeI* | Carbapenem, cephalosporin, diaminopyrimidine, fluoroquinolone, lincosamide, macrolide, penem, phenicol, rifamycin, tetracycline |
| Fosfomycin | *abaF* | Fosfomycin |
| Fluoroquinolone | *adeH* | Fluoroquinolone, tetracycline |
|  | *adeF* | Fluoroquinolone, tetracycline |
|  | *adeG* | Fluoroquinolone, tetracycline |
|  | *adeL* | Fluoroquinolone, tetracycline |
|  | *abaQ* | Fluoroquinolone |
| Sulfonamide | *sul1* | Sulfonamide |
| Phenicol | *catI* | Phenicol |
| Tetracycline | *tetA* | Tetracycline |
|  | *adeS* | Glycyclines, tetracyclines |
|  | *adeR* | Glycyclines, tetracyclines |
|  | *adeA* | Glycyclines, tetracyclines |
|  | *adeB* | Glycyclines, tetracyclines |
|  | *adeC* | Glycyclines, tetracyclines |
| Acridine dye | *abeM* | Acridine dye, fluoroquinolone, triclosan |
|  | *amvA* | Acridine dye, macrolide |
| Aminocoumarin | *abeS* | Aminocoumarin, macrolide |

**Table S5**. Antibiotic resistance profiles of *A. baumannii* ATCC BAA1605 identified by CARD.

| **Region** | **Completeness** | **Most common phage** | **Region length (Kb)** | **Region position** | **GC content (%)** |
| --- | --- | --- | --- | --- | --- |
| 1 | Intact | *Acinetobacter* YMC11/11/R3177 | 65.5 | 2784547-2850068 | 38.97 |
| 2 | Incomplete | *Psychromonas* pOW20 | 19 | 628224-647236 | 36.95 |
| 3 | Incomplete | *Cronobacter* ENT39118 | 16 | 711001-727091 | 37.23 |
| 4 | Incomplete | *Acinetobacter* YMC11/11/R3177 | 13.1 | 783023-796160 | 37.11 |
| 5 | Incomplete | *Acinetobacter* vB_AbaS_TRS1 | 32.9 | 3335679-3368591 | 38.53 |
| 6 | Incomplete | *Acinetobacter* Bphi-B1251 | 22.6 | 3371173-3393812 | 40.15 |

**Table S6**. Predicted prophage regions in *A. baumannii* ATCC BAA1605 using PHASTER.

| **Gene** | **Product** | **Start** | **End** |
| --- | --- | --- | --- |
| *nrdR* | Transcriptional repressor NrdR | 1780697 | 1781155 |
| *amtB_1* | Ammonia channel | 1781314 | 1782711 |
| *glnK* | Nitrogen regulatory protein P-II 2 | 1782769 | 1783107 |
| *comM_1* | Competence protein ComM | 1783680 | 1784537 |
| *bicA_2* | Bicarbonate transporter BicA | 1786710 | 1788197 |
| *lspA_1* | Lipoprotein signal peptidase | 1789908 | 1790420 |
| *zitB_2* | Zinc transporter ZitB | 1790424 | 1791320 |
| *merR1_1* | Mercuric resistance operon regulatory protein | 1791416 | 1791823 |
| *hin_1* | DNA-invertase Hin | 1792854 | 1793339 |
| *folP1* | Dihydropteroate synthase | 1794746 | 1795471 |
| *emrE_1* | Multidrug transporter EmrE | 1795465 | 1795812 |
| *ant1_1* | Streptomycin 3''-adenylyltransferase | 1795976 | 1796755 |
| *aacC1* | Gentamicin 3-N-acetyltransferase | 1797811 | 1798344 |
| *xerD_2* | Tyrosine recombinase XerD | 1798445 | 1799479 |
| *neo* | Aminoglycoside 3'-phosphotransferase | 1800885 | 1801700 |
| *hin_2* | DNA-invertase hin | 1803016 | 1803675 |
| *bla* | Beta-lactamase TEM | 1807474 | 1808334 |
| *tnpR* | Transposon Tn3 resolvase | 1808517 | 1809074 |
| *cat_2* | Chloramphenicol acetyltransferase | 1813230 | 1813889 |
| *tetA_1* | Tetracycline resistance protein, class C | 1817735 | 1819009 |
| *tetR* | Tetracycline repressor protein class A from transposon 1721 | 1819013 | 1819690 |
| *merR* | Mercuric resistance operon regulatory protein | 1820102 | 1820557 |
| *merC* | Mercuric transport protein MerC | 1821313 | 1821738 |
| *merA* | Mercuric reductase | 1821777 | 1823462 |
| *folP_1* | Dihydropteroate synthase | 1826493 | 1827332 |
|  | Single-stranded DNA-binding protein | 1828664 | 1829017 |
| *topA_2* | DNA topoisomerase 1 | 1829044 | 1831062 |
| *merR1_2* | Mercuric resistance operon regulatory protein | 1834689 | 1835096 |
| *arsC_2* | Arsenate reductase | 1835919 | 1836353 |
| *arsR1_2* | Arsenic resistance transcriptional regulator ArsR1 | 1836411 | 1836731 |
| *arsC_3* | Arsenate reductase | 1836738 | 1837211 |
| *acr3_2* | Arsenical-resistance protein Acr3 | 1837219 | 1838262 |
| *arsH* | NADPH-dependent FMN reductase ArsH | 1838268 | 1838972 |
|  | Glucosaminate ammonia-lyase | 1838990 | 1839943 |
| *tnsB_2* | Transposon Tn7 transposition protein TnsB | 1845205 | 1846632 |
| *comM_2* | Competence protein ComM | 1847467 | 1848150 |
| *gabR_2* | HTH-type transcriptional regulatory protein GabR | 1848187 | 1849605 |
| *cloR* | 4-hydroxy-3-prenylphenylpyruvate oxygenase/4-hydroxy-3-prenylbenzoate synthase | 1849717 | 1850493 |
| *dapA_3* | 4-hydroxy-tetrahydrodipicolinate synthase | 1850509 | 1851387 |
| *oprD* | Porin D | 1853159 | 1854475 |
| *ppa* | Inorganic pyrophosphatase | 1854582 | 1855109 |
| *fadD* | Long-chain-fatty-acid--CoA ligase | 1856164 | 1857843 |

**Table S7.** Genes found in Genomic Island 1 (1.78Mb to 1.86Mb) predicted by IslandViewer 4 in *A. baumannii* ATCC BAA1605 and their coordinates.

| **Gene** | **Product** | **Start** | **End** |
| --- | --- | --- | --- |
| *trmL* | tRNA (cytidine(34)-2'-O)-methyltransferase | 2891024 | 2891497 |
| *fldP_3* | Flavodoxin FldP | 2892043 | 2892603 |
| *plaP* | Low-affinity putrescine importer PlaP | 2892643 | 2894028 |
| *dhaT_1* | 1,3-propanediol dehydrogenase | 2894294 | 2895478 |
| *lipB* | Octanoyltransferase | 2896094 | 2896747 |
| *rpoD* | RNA polymerase sigma factor RpoD | 2897316 | 2899202 |
| *gltA_1* | Citrate synthase | 2902084 | 2903358 |
| *sdhC* | Succinate dehydrogenase cytochrome b556 subunit | 2904315 | 2904707 |
| *sdhD* | Succinate dehydrogenase hydrophobic membrane anchor subunit | 2904707 | 2905072 |
| *sdhA* | Succinate dehydrogenase flavoprotein subunit | 2905085 | 2906920 |
| *sdhB* | Succinate dehydrogenase iron-sulfur subunit | 2906935 | 2907645 |
| *sucA* | 2-oxoglutarate dehydrogenase E1 component | 2908214 | 2911054 |
| *sucB* | Dihydrolipoyllysine-residue succinyltransferase component of 2-oxoglutarate dehydrogenase complex | 2911054 | 2912250 |
| *lpdG* | Dihydrolipoyl dehydrogenase | 2912313 | 2913746 |
| *sucC* | Succinate--CoA ligase [ADP-forming] subunit beta | 2913894 | 2915060 |
| *sucD* | Succinate--CoA ligase [ADP-forming] subunit alpha | 2915075 | 2915965 |
| *trpS2* | Tryptophan--tRNA ligase 2 | 2918130 | 2919143 |
| *yedI* | Inner membrane protein YedI | 2919350 | 2920273 |
| *nhaP* | Na(+)/H(+) antiporter NhaP | 2921440 | 2922942 |
| *capA* | Capsule biosynthesis protein CapA | 2946287 | 2947024 |
|  | DNA base-flipping protein | 2947819 | 2948148 |
| *teaD* | TRAP-T-associated universal stress protein TeaD | 2948204 | 2948641 |
| *cat_3* | Chloramphenicol acetyltransferase | 2948762 | 2949394 |
| *greA* | Transcription elongation factor GreA | 2949473 | 2949949 |
| *carB* | Carbamoyl-phosphate synthase large chain | 2950037 | 2953267 |
| *carA* | Carbamoyl-phosphate synthase small chain | 2953282 | 2954421 |
|  | RNA-binding protein | 2955931 | 2956254 |
| *rlmE* | Ribosomal RNA large subunit methyltransferase E | 2956453 | 2957103 |
| *ftsH* | ATP-dependent zinc metalloprotease FtsH | 2957238 | 2959133 |
| *folP_2* | Dihydropteroate synthase | 2959265 | 2960116 |
| *phoD* | Alkaline phosphatase D | 2961267 | 2963030 |
| *folD* | Bifunctional protein FolD protein | 2963453 | 2964301 |
| *prmA_1* | Ribosomal protein L11 methyltransferase | 2964542 | 2965210 |
| *kgtP_2* | Alpha-ketoglutarate permease | 2965771 | 2967090 |
| *glpK* | Glycerol kinase | 2967477 | 2968985 |

**Table S8**. Genes found in Genomic Island 2 (2.89Mb to 2.97Mb) predicted by IslandViewer 4 in *A. baumannii* ATCC BAA1605 and their coordinates.

| **Similarity** | **Name** |
| --- | --- |
| 1 | MK386683.1 *Acinetobacter baumannii* strain ABAY14012 plasmid pABAY14012_4D, complete sequence |
| 0.998 | NZ_CP027244.1 *Acinetobacter baumannii* strain WCHAB005078 plasmid p2_005078, complete sequence |
| 0.994 | CM009043.2 *Acinetobacter baumannii* strain ZQ5 plasmid p2ZQ5, complete sequence, whole genome shotgun sequence |
| 0.994 | CP002523.1 *Acinetobacter baumannii* TCDC-AB0715 plasmid p1ABTCDC0715, complete sequence |
| 0.994 | CP007578.1 *Acinetobacter baumannii* AC30 plasmid pAC30a, complete sequence |
| 0.994 | KU869528.1 *Acinetobacter baumannii* strain A297(RUH875) plasmid pA297-2, complete sequence |
| 0.994 | NC_022565.1 *Acinetobacter baumannii* 107m plasmid p1ABIBUN complete genome |
| 0.994 | NZ_CP008850.1 *Acinetobacter baumannii* strain AC29 plasmid pAC29a, complete sequence |
| 0.994 | NZ_CP010782.1 *Acinetobacter baumannii* strain A1 plasmid pA1-1, complete sequence |
| 0.994 | NZ_CP020576.1 *Acinetobacter baumannii* strain SSA12 plasmid pSSA12_2, complete sequence |

**Table S9.** Top 10 plasmids and their similarity with *A. baumannii* ATCC BAA1605 plasmid using sourmash search-containment method against PLSDB database.

| Start | 3171848 |
| --- | --- |
| End | 3174997 |
| Sequences of repeats | GTTCATGGCGGCATACGCCATTTAGAAA |
| Length of repeats | 28 |
| Spacer counts | 52 |

**Table S10**. Summary of CRISPR array identified in chromosome of *A. baumannii* ATCC BAA1605.

| **Gene name** | **Start** | **End** |
| --- | --- | --- |
| *cas1* | 3163579 | 3164544 |
| *cas3-cas2* | 3164541 | 3167915 |
| *csy1* | 3167931 | 3168752 |
| *csy2* | 3169179 | 3170063 |
| *csy3* | 3170089 | 3171102 |
| *cas6* | 3171105 | 3171716 |

**Table S11**. Subtype I-F *cas* genes identified in the chromosome of *A. baumannii* ATCC BAA1605 and their coordinates.


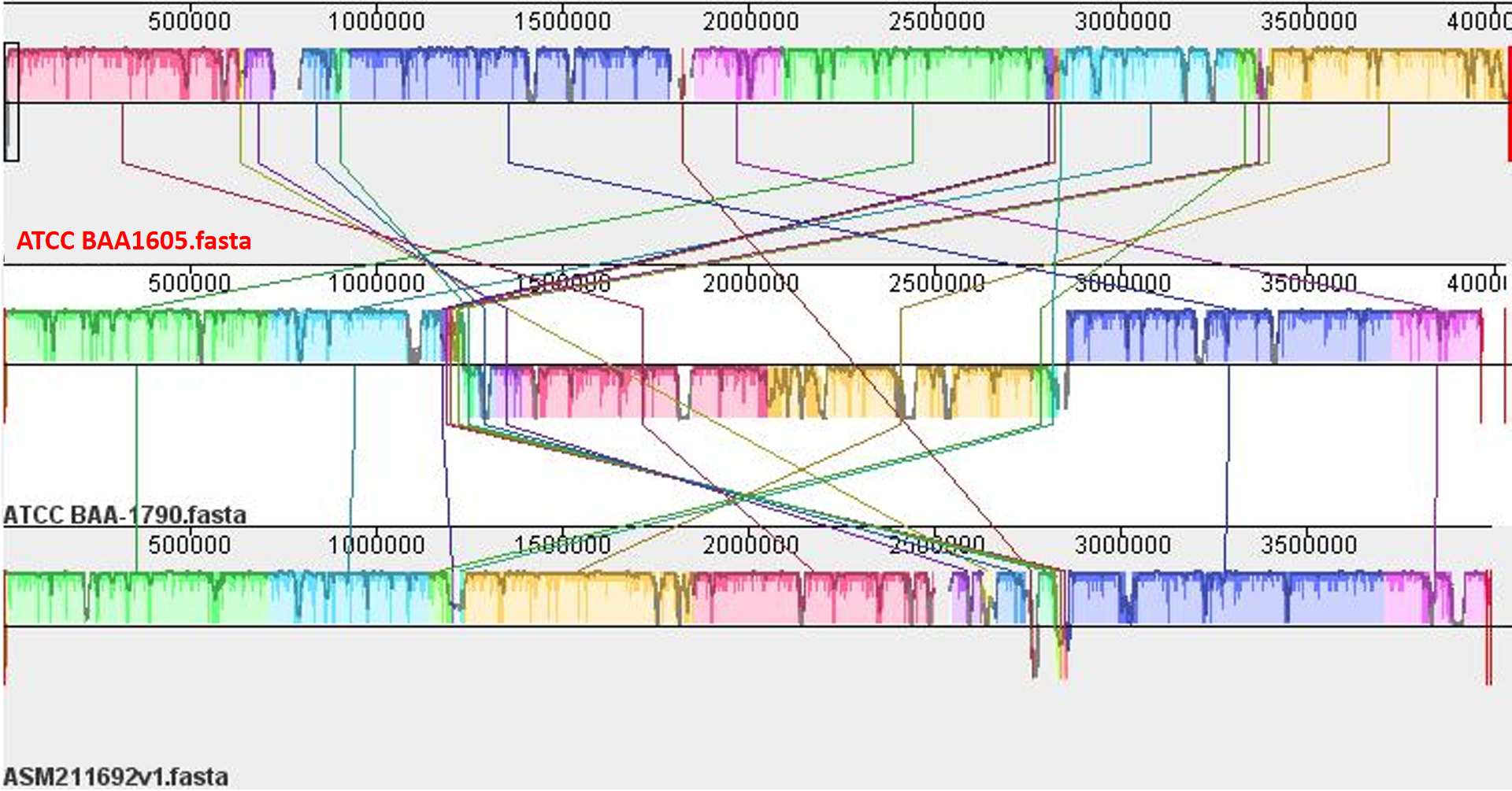


**Figure S1.** Whole genome alignments of *A. baumannii* ATCC BAA1605 (top), *A. baumannii* ATCC BAA-1790 (middle) and *A. baumannii* ASM211692v1 (bottom) using Mauve. Each coloured blocks depicts the homologous sites of sequence that aligned to part of another genomes.


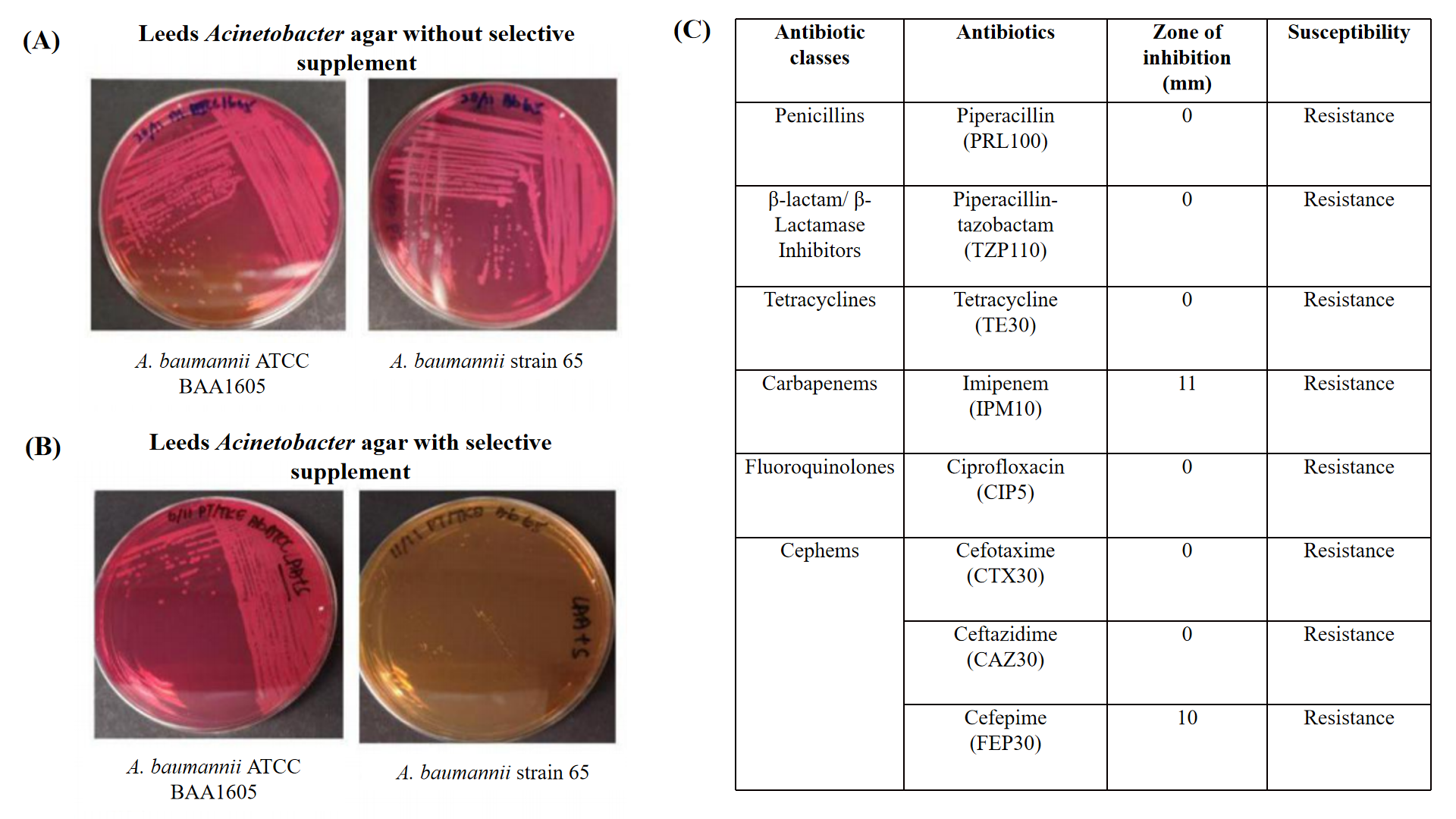


**Figure S2.** Identification and antibiotic resistance profiling of *A. baumannii* ATCC BAA1605. MDR *A. baumannii* ATCC BAA1605 and antibiotic-sensitive *A. baumannii* strain 65 (control) cultured on Leeds *Acinetobacter* medium either without selective supplement (A) or with antibiotics supplementation (B). (C) Antibiotic susceptibility profiling of *A. baumannii* ATCC BAA1605 using disk diffusion method.
